# Abamectin Causes Neurotoxicity in Zebrafish Embryos

**DOI:** 10.3390/ijms26010349

**Published:** 2025-01-03

**Authors:** Hongying Zhang, Yulong Liu, Yukun Huang, Kaiwen Zhao, Tingting Yu, Youjuan Wu, Zijia Yin, Meifeng Li, Dongming Li, Lihua Fan, Xiaowen Xu, Chengyu Hu, Shanghong Wang

**Affiliations:** 1 School of Life Science, Nanchang University, Nanchang 330031, China18807080298@163.com (Y.H.);; 2 Institute of Pathogenic Microorganism, College of Bioscience and Engineering, Jiangxi Agricultural University, Nanchang 330045, China; 3 Fuzhou Medical College, Nanchang University, Fuzhou 344000, China; dongmingli2005@163.com; 4 Chongqing Research Institute of Nanchang University, Chongqing 402660, China

**Keywords:** insecticide, neurodevelopmental toxicity, oxidative stress, apoptosis, zebrafish embryos

## Abstract

Abamectin is an insecticide, miticide and nematicide that has been extensively used in agriculture for many years. The excessive use of abamectin inevitably pollutes water and soil and might even cause adverse effects on aquatic biota. However, it is currently unclear how abamectin exposure causes neurotoxicity in aquatic organisms. Herein, the early neural system development was assessed in zebrafish embryos following abamectin exposure. After treatment with a concentration gradient of abamectin (0.055, 0.0825, 0.11 mg/L), the survival rate, average heart rate, pericardial edema area and yolk sac edema were all documented in zebrafish embryos (96 hpf). It was found that after abamectin exposure, embryonic brain development was impaired, and motor behaviors were also affected. The fluorescence intensity was reduced in the transgenic embryos (*Eno2: GFP*). The activities of acetylcholinesterase (AChE) and ATPase were decreased, and the expression of neurodevelopment-related genes, such as *sox10*, *gap43*, *grin1b*, *abat*, *gad1b*, *grin2b*, *nestin* and *glsa*, were all inhibited in zebrafish embryo treatment with abamectin. Furthermore, the reactive oxygen species (ROS) were triggered upon exposure to abamectin in zebrafish embryos along with the accumulation of ROS, eventually resulting in neuroapoptosis in the developing embryonic brain. In conclusion, neurodevelopmental toxicity was caused by oxidative stress-induced apoptosis in zebrafish embryos following abamectin exposure.

## 1. Introduction

Over the past several decades, pesticides have been widely used in agriculture around the world. Inevitably, pesticide residues gradually accumulate in soils, rivers and lakes [1], threatening the survival of many terrestrial and aquatic organisms, such as beneficial insects, fish, shrimp, crabs, etc.

Abamectin belongs to the avermectin family. The presently commercialized avermectin, which is produced by the fermentation of *Streptomyces avermitilis*, has abamectin as the major insecticidal ingredient. As a broad-spectrum insecticide, abamectin is abundantly used to fight against harmful insects, mites, fungi and bacteria in agriculture [2,3,4]. Pure abamectin is a white crystalline powder that is more easily dissolved in organic solvents than in water. Abamectin can be detected in a variety of samples, such as feces (0.2 mg·kg^−1^), soil–feces mixture (0.2 mg·kg^−1^) and the milk of cattle (0.005 mg·kg^−1^) [5,6,7]. Depending on the different environmental factors, the half-life of abamectin ranges from as quickly as 12 h to as long as 23 days. In fact, it accumulates in organisms before its degradation. Nevertheless, following its widespread use, abamectin may be accumulated in various kinds of organisms, giving rise to high toxicity to many beneficial mites, earthworms, aquatic animals, etc. The highest and lowest concentrations of abamectin in soil are 45.5 μg·kg^−1^ and 0.2 μg·kg^−1^, respectively. In animals, such as in bees, the residue level of abamectin ranges from 32 μg·kg^−1^ to 43.0 μg·kg^−1^. In plants, particularly in leaves, the residue level ranges from 0.3 μg·kg^−1^ to 2.4 μg·kg^−1^. Additionally, the residue level of abamectin in groundwater ranges from 3.8 μg·kg^−1^ to 15 μg·kg^−1^ [8].

It was reported that *Phytoseius plumifer* was totally killed after exposure to abamectin at 0.00018 µg·cm^−2^ within 24 h [9]. Abamectin shows toxicity to earthworms at 0.25 mg·kg^−1^, and once abamectin levels exceed 5 mg·kg^−1^, earthworms lose their reproductive capacity [10]. After exposure to abamectin, the brain of king pigeon is impaired, and an inflammatory response is induced as well [11]. However, relatively few studies are focused on the potential toxicity of abamectin to aquatic animals. After exposure to high concentrations of abamectin, the conduction of nervous impulses is continuously increased, eventually bringing about abnormal behaviors in crayfish [12]. The apoptosis is activated by abamectin residues in carp gill cells through exogenous pathways [13]. Avermectin can induce neurotoxicity in carp by disrupting blood–brain barrier structures and producing oxidative stress, inflammation and apoptosis [14]. Abamectin is not only harmful to fish, but also to algae, water fleas and bacteria as well. For example, abamectin can cause anxiety and open-mouth death in *Rutilus caspicus* [15]. It is well known that abamectin is harmful to aquatic animals but its mechanism is still undetermined. Hence, it is urgent to explore the potential environmental risks involved in abamectin exposure.

For a long time, zebrafish have been used as an excellent model organism to assess the toxicity of pollutants or pesticides [16]. In this study, to investigate the neurotoxicity of abamectin to aquatic organisms, the morphological changes and motor behaviors were both examined in zebrafish embryos upon abamectin exposure; meanwhile, the expression patterns of some nerve-related genes, oxidative stress and apoptosis genes were all detected. These results suggested that neurodevelopment is severely impaired in zebrafish embryos after exposure to abamectin.

## 2. Results

### 2.1. Abamectin Causes Developmental Toxicity in Zebrafish Embryos

**Figure 1:**
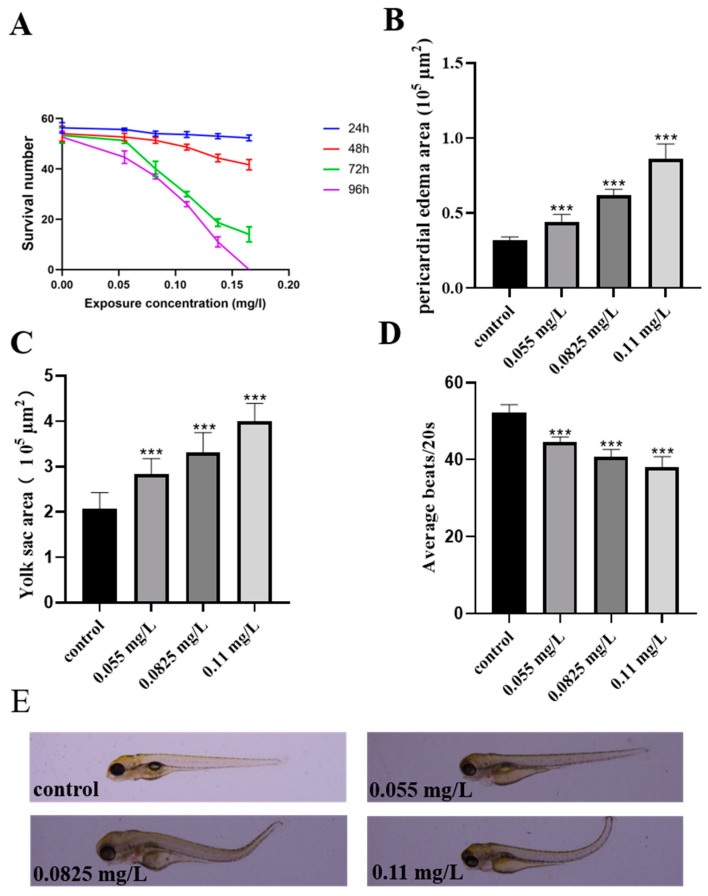
Exposure to abamectin induces severe developmental toxicities in zebrafish embryos. (**A**) The survival rate of zebrafish embryos following abamectin exposure at different concentrations during the exposure period (24 hpf to 96 hpf). (**B**–**D**) The toxicities of abamectin to pericardial edema area, yolk sac area and heart rate in zebrafish embryos. (**E**) Phenotype of zebrafish embryos after exposure to different concentrations of abamectin at 96 hpf. Asterisks denote significant differences in comparison with the control. Data were expressed as mean ± SD of three independent experiments (n = 60; *** *p* < 0.001; “n” represented the number of embryos in each experiment).

The molecular structure of abamectin is shown in Figure S1. The results indicated that the survival rate of zebrafish embryos was decreased in an abamectin concentration-dependent manner at various time points (24–96 hpf), and the LC50 for zebrafish embryos exposed to abamectin at 96 hpf was 0.11 mg/L (Figure S2 and Figure 1A). In view of this, a concentration gradient of abamectin (0.055, 0.0825 and 0.11 mg/L) was used in our subsequent experiments in this study.

Upon exposure to abamectin, the pericardial edema area and yolk sac area were both increased in zebrafish embryos (Figure 1B,C), while heart rate was decreased in embryos (Figure 1D). Therefore, these results indicated that abamectin indeed causes developmental toxicity of zebrafish embryos.

### 2.2. Abamectin Damages Neurodevelopment in Zebrafish Embryos

**Figure 2:**
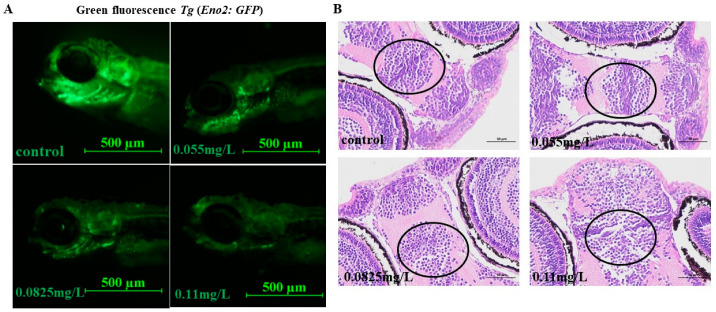
Abamectin causes damage to the brain of zebrafish larvae. (**A**) *Tg (Eno2: GFP)* transgenic zebrafish embryos were used to analyze neurotoxicity in zebrafish embryos. All embryos were separately exposed to abamectin at different concentrations (0.055 mg/L, 0.0825 mg/L and 0.11 mg/L) and collected at 96 hpf. (**B**) The transverse slices of the brain were prepared using a hematoxylin eosin staining method. The abnormal part of the brain was marked with black ovals. All embryos were separately exposed to abamectin at concentrations of 0.055 mg/L, 0.0825 mg/L and 0.11 mg/L. At 96 hpf, ten embryos were randomly selected from each group and stained in HE reagent. These experiments were representatives of three independent replicates.

To further assess the toxicity of abamectin in zebrafish embryos, the transgenic zebrafish *Tg* (*Eno2: GFP*) was used in this study. *Eno2* is a neurodevelopment-related gene. After exposure to abamectin, the fluorescence intensity (*Eno2: GFP*) of embryos was obviously reduced, suggesting that abamectin caused damage to the nervous system (Figure 2A).

In addition, HE stains showed that the overall brain of zebrafish was impaired. In detail, the forebrain morphology was gradually deformed, the number of cells was decreased and the intercellular space was increased, indicating that abamectin causes damage in the brain (Figure 2B).

### 2.3. Abamectin Undermines the Behavior Ability of Zebrafish Larvae

**Figure 3:**
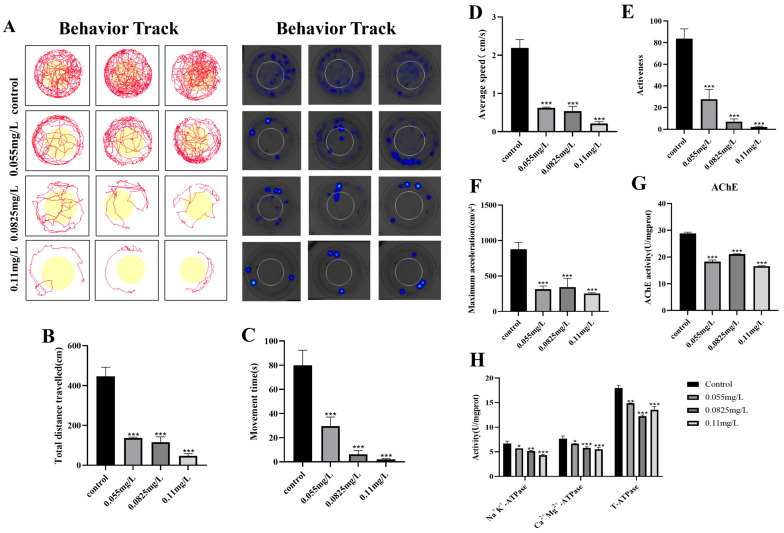
The effects of behavioral indicators caused by abamectin. (**A**) All embryos were separately exposed to abamectin at different concentrations (0.055 mg/L, 0.0825 mg/L and 0.11 mg/L) up to 96 hpf, and the motion trajectories of zebrafish larvae in each group were separately recorded. The experiments were representatives of three independent replicates. (**B**–**F**) The data of the total distance, movement time, average speed, activity and maximum acceleration were also recorded. Data were expressed as mean ± SD of three independent experiments (n = 6; * *p* < 0.05, ** *p* < 0.01 and *** *p* < 0.001; “n” represents the number of embryos at each experiment). (**G**,**H**) All embryos were exposed to abamectin (0.055 mg/L, 0.0825 mg/L and 0.11 mg/L) until 96 hpf. The activities of AChE and ATPase were detected and calculated. The data were expressed as the mean ± SD of three independent experiments (n = 30; * *p* < 0.05, ** *p* < 0.01 and *** *p* < 0.001; “n” represents the number of embryos in each experiment). Asterisks denote significant differences in comparison with control.

The behavior ability was frequently used to evaluate the neurodevelopmental defects in zebrafish embryos. The motion trajectory of zebrafish larvae at 96 hpf was decreased in abamectin-treated groups (Figure 3A). The other behavior index was also recorded, such as average speed, moving time, moving distance activeness and maximum acceleration, which suggested that the behavioral abilities of zebrafish larvae were notably impaired following exposure to 0.055, 0.0825 and 0.11 mg/L of abamectin (Figure 3B–F). Moreover, the activities of AChE and ATPase were inhibited under treatment with abamectin (Figure 3G,H). The results suggested that abamectin not only damages neurodevelopment but also undermines the behavioral ability of zebrafish larvae.

### 2.4. Abamectin Inhibits the Expression of Neurodevelopment-Related Genes

**Figure 4:**
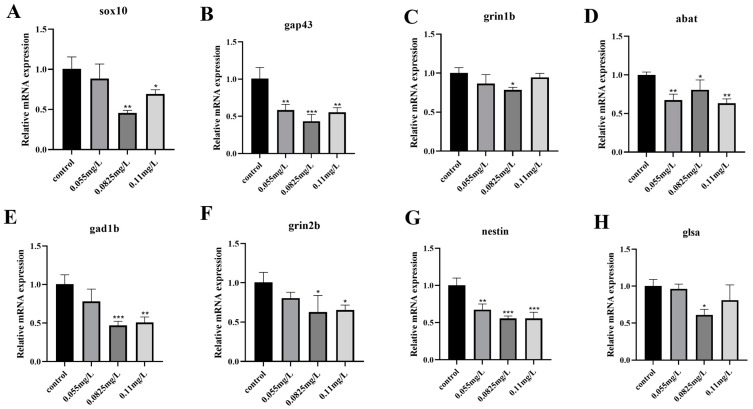
The reduction in the expression of neurodevelopment-related genes caused by abamectin. (**A**–**H**) All embryos were separately exposed to abamectin at different concentrations (0.055 mg/L, 0.0825 mg/L and 0.11 mg/L) and collected at 96 hpf. qRT-PCR was used to detect the expression profiles of neurodevelopment-related genes (*sox10*, *gap43*, *grin1b*, *abat*, *gad1b*, *grin2b*, *nestin* and *glsa*). The data were expressed as the mean ± SD of three independent experiments (n = 30; * *p* < 0.05, ** *p* < 0.01 and *** *p* < 0.001; “n” represents the number of embryos in each experiment). Asterisks denote significant differences in comparison with the control.

To explore the molecular mechanism of neurotoxicity caused by abamectin, some neurodevelopment-related genes, such as *sox10*, *gap43*, *grin1b*, *abat*, *gad1b*, *grin2b*, *nestin* and *glsa*, were detected (Figure 4A–H). The expression of these genes was all inhibited following abamectin exposure, indicating that abamectin may suppress the development of neurons and synaptic transmission in zebrafish embryos.

### 2.5. Abamectin Activates Oxidative Stress of Zebrafish Embryos

**Figure 5:**
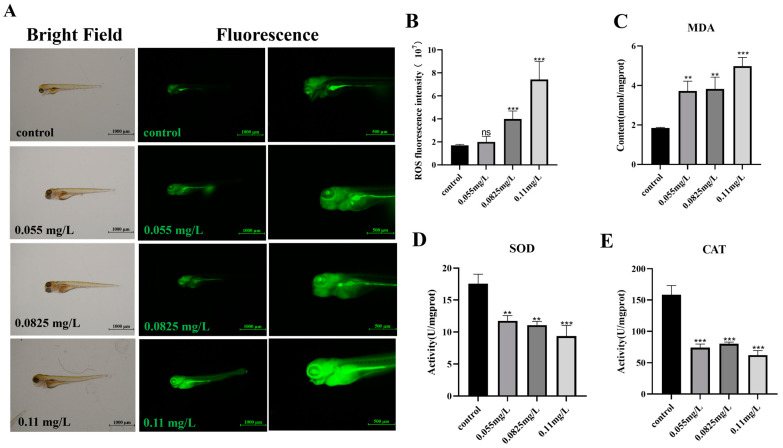
The effects of oxidative stress indicators induced by abamectin. (**A**) Zebrafish embryos were separately exposed to abamectin at different concentrations (0.055 mg/L, 0.0825 mg/L and 0.11 mg/L), and then ROS was detected in zebrafish embryos with fluorescent probes (DCFH-DA) at 96 hpf. The experiments were representatives of three independent replicates. (**B**) Fluorescent intensity was calculated using NIS-element D software. Data were expressed as mean ± SD of three independent experiments (n = 10; ns *p* > 0.05, *** *p* < 0.001; “n” represents the statistical number of embryos in each experiment). (**C**–**E**) The activities of CAT, SOD and the content of MDA were detected in zebrafish embryos. Data were expressed as mean ± SD of three independent experiments (n = 30; ** *p* < 0.01 and *** *p* < 0.001; “n” represents the number of embryos in each experiment). Asterisks denote significant differences in comparison with control.

ROS induction gradually increased together with the increased concentration of abamectin exposure (Figure 5A). The fluorescence intensity of 2,7-dichlorofluorescein diacetate (DCFH-DA) in each group was calculated, and the results showed a significant increase of ROS accumulation in larvae exposed to higher concentrations of abamectin (Figure 5B). In addition, the amount of malondialdehyde (MDA) was increased while the activities of superoxide dismutase (SOD) and catalase (CAT) were decreased (Figure 5C–E). These results indicated that oxidative stress is activated in zebrafish embryos under stimulation with abamectin.

### 2.6. Abamectin-Induced Oxidative Stress Increases Apoptotic Cells in the Brain Area

**Figure 6:**
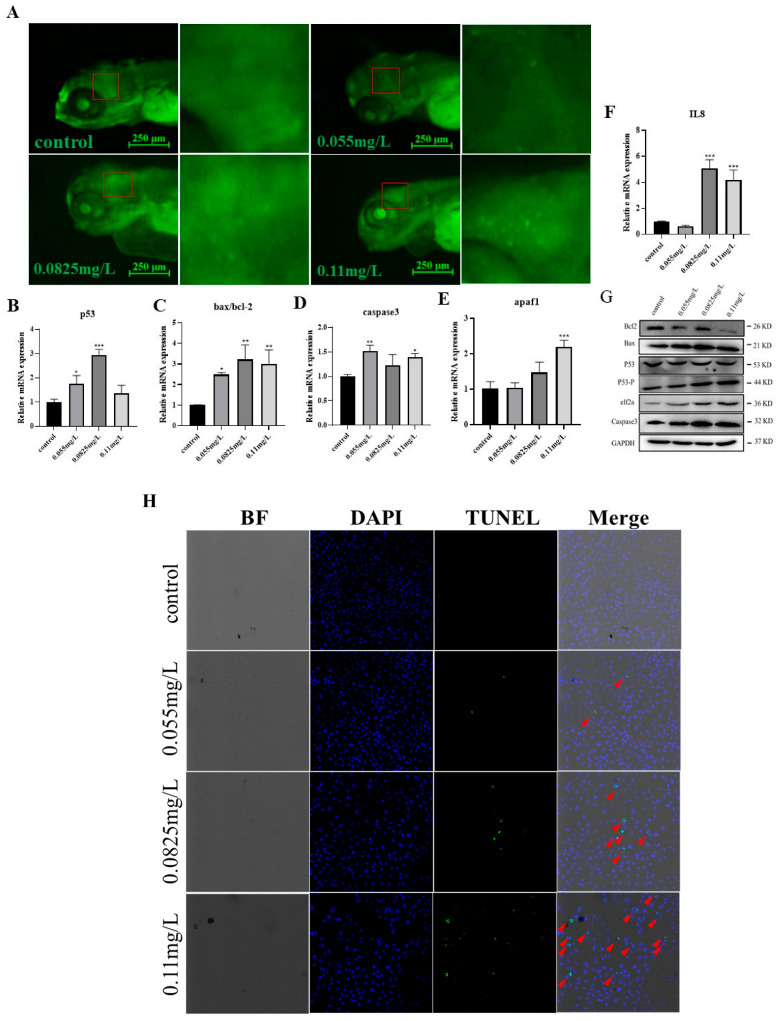
Abamectin exacerbates apoptosis in zebrafish larvae and ZF4 cells. (**A**) Zebrafish embryos were separately exposed to abamectin at different concentrations (0.055 mg/L, 0.0825 mg/L and 0.11 mg/L), and the embryos were stained with acridine orange at 96 hpf; apoptotic cells are indicated by bright green dots, and the image on the right is a red box on the left for enlarged view. The experiments were represented by three independent replicates. (**B**–**F**) Some apoptotic and inflammation-related genes (*p53*, *bcl2*, *bax*, *caspase3*, *apaf1* and *IL8*) were detected by qRT-PCR. Panel C showed the expression ratios of *bcl2* and *bax*. (**G**) Western blot for some apoptosis-related genes (bcl2, bax, p53, p53-p, caspase3 and eIF2α). (**H**) Zebrafish ZF4 cells were exposed to different concentrations (0.055 mg/L, 0.0825 mg/L and 0.11 mg/L) of avermectin, TUNEL staining and detection of apoptotic cells by fluorescence microscopy. The red triangle indicated the phenomenon of apoptosis. Data were expressed as mean ± SD of three independent experiments (n = 30; * *p* < 0.05, ** *p* < 0.01 and *** *p* < 0.001; “n” represents the number of embryos in each experiment). Asterisks denote significant differences in comparison with control.

Excessive oxidative stress often results in apoptosis. To confirm whether abamectin-induced oxidative stress activates apoptosis in zebrafish embryos, Acridine Orange (AO) staining was performed in this study. The results showed that abamectin increased apoptosis (granular and bright green dots) in the embryonic brain (Figure 6A). Moreover, the expressions of apoptosis-related and inflammatory factor genes such as *bax*, *caspase3*, *p53*, *apaf1* and *IL8* were all increased after abamectin exposure (Figure 6B–G). TUNEL stain also indicated that abamectin induced apoptosis in ZF4 cells (Figure 6H). These results showed that apoptosis was triggered in the zebrafish embryonic brain upon exposure to abamectin.

## 3. Discussion

With the widespread application of abamectin, its accumulation in the environment and organisms is likely to rise. Therefore, it is urgent to uncover the potential risks associated with abamectin. This study aims to evaluate the toxicity of abamectin in zebrafish embryos. Our preliminary experiment explored the toxic concentration for zebrafish embryos (Figure S2), indicating that the concentration of exposure was below 0.055 mg/L, and the lowest observed effect (LOEC) was shown; when the exposure concentration exceeded 0.11 mg/L, it caused more than half of all fatalities. Thus, a concentration gradient of abamectin (0.055, 0.0825 and 0.11 mg/L) was used in the subsequent experiments. It was also found that heart rates were decreased while the yolk sac area and pericardial edema area were increased after a series of concentrations of abamectin exposure (0.055, 0.0825 and 0.11 mg/L) (Figure 1). Therefore, abamectin can cause severe developmental toxicity to zebrafish embryos.

It is reported that abamectin causes neurotoxicity in *Panonychus citri* by inhibiting glutamate synthesis and γ-aminobutyric acid-gated chloride channels, blocking neurotransmission and interfering with neuromuscular synapses [17]. To verify its toxicity to aquatic organisms, transgenic zebrafish embryos *Tg* (*Eno2: GFP*) were used to explore whether neurotoxicity can be induced in zebrafish embryos upon exposure to abamectin. It is confirmed that the *eno2* gene (namely, *enolase-2*) is specifically expressed in mature neurons throughout the neural axis [18,19]. Our results showed that fluorescence intensity was decreased in the zebrafish embryonic brain after abamectin exposure (Figure 2A). Moreover, cellular damages were observed in the brain area using HE staining (Figure 2B). So, exposure to abamectin leads to severe neurotoxicity in zebrafish larvae. It is important to clarify whether the neurotoxicity by abamectin affects the behavior of zebrafish larvae. Behavioral analysis is considered to be an effective way to evaluate neurotoxicity [20]. AChE is a significant hydrolase of the acetylcholine neurotransmitter and its activity is involved in the function of the nervous system [21]. Na^+^K^+^-ATPases and Ca^2+^Mg^2+^-ATPases control the transportation of AChE. Therefore, the activities of AChE and Na^+^K^+^-ATPase and Ca^2+^Mg^2+^-ATPase are often used as the markers to evaluate neurotoxic exposure [22]. Our results indicated that motor behaviors were inhibited and the activities of AChE and ATPase were both decreased in zebrafish embryos (Figure 3), implying that neurotoxicity was elicited in zebrafish embryos after exposure to abamectin. Similarly, the escape capacity and swimming ability of mice are reduced by abamectin due to the alteration of the expression of HSP70 and P-gp [23]. Abamectin inhibits ATPase phosphohydrolase activity, which impairs the bioenergetics of mitochondria in rat livers [24].

To further investigate the mechanism of neurotoxicity caused by abamectin, we examined several neurodevelopment-related genes. Some neurogenesis-related genes, such as *grin1b* and *gap43*, are involved in the regulation of growth and development of neuronal synapses [25]. The expression of *gad1b* and *sox10* is essential during the development of neurons [26,27]. In the process of neuronal transmission, *grin1b* and *glsa* are critical in synaptic transmission [28,29]. *Abat* and *gad1b* are necessary for the neurotransmitter system [30]. *Nestin* is associated with the production of neurons and glial cells [31]. *Sox10*, *gad1b*, *nestin* and *gap43* play important roles in the growth and development of neurons [25,27,28,31]. These genes, including *grin1b*, *abat* and *glsa*, are critical for synaptic transmission [29,30]. Therefore, these neurodevelopmental genes (*sox10*, *gap43*, *grin1b*, *abat*, *gad1b*, *grin2b*, *nestin* and *glsa*) were all tested following exposure to abamectin. The results of qRT-PCR showed that the expression profiles of these genes were all decreased (Figure 4), suggesting that the expression of neurodevelopmental genes may be inhibited due to abamectin-induced neurotoxicity in zebrafish embryos. The inhibition of neurogenic expression not only impairs the development of neuronal synapses, neurons and neurotransmitter systems but also reduces the production of glial cells, thereby disrupting the smooth transmission of signals at synapses.

The decreased expressions of neurodevelopmental genes are also associated with oxidative stress [32]. Oxidative stress and apoptosis may be associated with the production of ROS that occur following the caspase cascade via the mitochondrial and death receptor pathways [33]. Environmental pollutants can cause oxidative stress, which is caused by an imbalance between oxidants and antioxidants at the cellular level. The accumulation of ROS facilitates the occurrence of oxidative stress. The excessive ROS causes damage to DNA structure and changes the activities of CAT, SOD and MDA [34]. MDA is a major product of lipid peroxidative damage [35,36]. Both SOD and CAT belong to antioxidant enzymes [37,38]. This study showed that abamectin significantly induced ROS in the zebrafish embryonic brain (Figure 5A,B). Moreover, abamectin increased the concentration of MDA but decreased the activities of CAT and SOD (Figure 5C–E). Similarly, abamectin increases ROS, decreases the antioxidant defense and induces hepatocytic apoptosis in *Schizothorax prenanti* [39].

The continuous activation of oxidative stress causes damage to cell stability and even induces apoptosis [40]. In this study, AO staining showed that abamectin induced a number of apoptotic cells (green dots) in the zebrafish embryonic brain, and then activated the expressions of apoptosis-related genes (*bax*, *p53* and *caspase3*) and inflammatory factors (*apaf1* and *IL-8*) as well (Figure 6). Similarly, abamectin activates caspase-3 and caspase-9 through a mitochondria-mediated pathway, inducing apoptosis in both human (HepG2) and insect (Sf9) cells [33]. As a consequence, our study demonstrated that abamectin is neurotoxic to zebrafish embryos, indicating that abamectin-induced neurotoxicity may be due to the oxidative stress-activated apoptosis in the zebrafish embryonic brain. Our research provided a new insight into the evaluation of the neurotoxicity of abamectin in aquatic organisms.

## 4. Materials and Methods

### 4.1. Reagents and Zebrafish Strains

Abamectin (CAS: 71751-41-2, analytically pure) was purchased from Aladdin (Aladdin, Beijing, China) and dissolved in DMSO at a concentration of 1.1 g/L (0.055 g powder of abamectin dissolved in 50 mL of DMSO). In this study, abamectin solutions were prepared at concentrations ranging from 0.055 to 0.165 mg/L (0.055, 0.0825, 0.11, 0.1375, 0.165 mg/L). RNA extraction and cDNA reverse-transcription kits were bought from Tiangen (Tiangen, Beijing, China) and TaKaRa (TaKaRa, Tokyo, Japan), respectively. Kits of superoxide dismutase (SOD), malondialdehyde (MDA), catalase (CAT), Na^+^K^+^-ATPase and Ca^2+^Mg^2+^-ATPase, ROS and AChE were all from Nanjing Jiancheng Biotechnology Co., Ltd. (Nanjing Jiancheng Bioengineering Institute, Nanjing, China). TUNEL BrightGreen Apoptosis Detection Kit was from Novozan Biotechnology Co., Ltd. (Vazyme, Nanjing, China). N-phenylthiourea (PTU) (Sigma, St. Louis, MO, USA) was used to inhibit the production of melanin in zebrafish embryos.

ZF4 cells were gifted from Prof. Zou Jun of Shanghai Ocean University. AB strain wild-type and *Tg* (*Eno2: GFP*) Zebrafish were from Nanjing EzeRinka Biotechnology Co., Ltd. (EzeRinka, Nanjing, China). The feeding method was to raise all fish in a recirculating water system (ESEN, Beijing, China). The temperature was maintained at 28 °C ± 0.5 °C, and Artemia was fed twice a day on a 14 h light:10 h dark cycle. The pH of the circulating water was kept in the range of 7.0~7.5, and the conductivity was kept at 500~700 μs/cm. All fish were fed in the same way as described in our previous study [41].

### 4.2. Abamectin Exposure and Morphological Observation of Zebrafish Embryos

Every 60 healthy embryos at 6 h post-fertilization (hpf) were separately cultured in 75 cm^2^ dishes. Zebrafish embryos were separately exposed to different concentrations of abamectin (0.055, 0.0825, 0.11, 0.1375 and 0.165 mg/L) at 6–96 hpf. The culture medium containing abamectin was replaced every 24 h. The control group was treated with 0.01% DMSO (2 μL of DMSO added in 20 mL embryo culture medium). It is acceptable that 0.01% DMSO is often used as control and shows non-toxic to zebrafish embryos [42]. After exposure, the survival rate, average heart rate, pericardial edema area and yolk sac edema area of the embryos at 96 hpf were seriously documented. Nikon image software (NIS-Elements D NIS Viewer-5.21.00) was used to measure the area of pericardial edema and yolk sac edema. Transgenic zebrafish *Tg* (*Eno2: GFP*) embryos were used to observe neurodevelopment. The average heart rate was measured with a timer using a stereo microscope (Nikon, Tokyo, Japan), and the phenotypic malformations of zebrafish larvae were also examined under the same stereo microscope. The fluorescence of *Tg* (*Eno2: GFP*) was photographed using SMZ800-FL fluorescence microscope (Nikon, Tokyo, Japan). The experiment was independently repeated three times and the embryos of different replications were from different parents.

### 4.3. Total RNA Extraction and Quantitative RT-PCR

RNA simple Total RNA Kit (Tiangen, Beijing, China) was used to isolate total RNA of zebrafish embryos (30 embryos per group). Firstly, zebrafish embryos exposed to various concentrations of abamectin were washed three times with embryo culture medium; lysate RZ was added and fully ground and lysed; the supernatant was centrifuged; chloroform was added; the supernatant was taken away once more. Absolute ethanol was added to precipitate RNA. Next, the samples were washed with deproteinization solution RD and rinse solution RW, then eluted with RNase-Free ddH_2_O. Finally, 2 μg of RNA was used to synthesize first-strand cDNA. The relative expressions of some genes involved in neurodevelopment, apoptosis or inflammation (*sox10*, *gap43*, *grin1b*, *abat*, *gad1b*, *grin2b*, *nestin*, *glsa*, *p53*, *bax*, *bcl2*, *caspase3*, *apaf1* and *IL8*) were detected. *β-actin* was used as an internal control gene. The specific primers used in qRT-PCR are listed in Table 1.

### 4.4. Hematoxylin Eosin (HE) Stain

After exposure to abamectin, 10 embryos were collected at 96 hpf and fixed in 4% paraformaldehyde overnight. The protocols of slicing and HE staining are as follows: Firstly, the fixed zebrafish were randomly selected, rinsed and successively dehydrated in 70%, 80%, 90%, 95% and 100% ethanol. The zebrafish were transparent in xylene and then transferred into paraffin wax for permeabilization; finally, the paraffin-embedded samples were sectioned into 4 µm of slices. HE staining was carried out as follows: exhibition, patching, re-hydration, dyeing, dehydration, transparency and mounting [43]. The prepared slices were photographed under a microscope in which the nucleus was shown in blue and the cytoplasm in red. Finally, the samples were photographed under an upright fluorescence microscope (Nikon, Tokyo, Japan).

### 4.5. Behavioral Analysis and Detection of AChE and ATPase Activity

Six embryos in each group were randomly transferred to 24-well plates (one embryo per well), followed by adaptation to environment for 10 min. Subsequently, 5 min of behavioral data was separately collected using Noldus Danio Vision system (Noldus, Wageningen, The Netherlands), including movement tracking, movement time, distance, average speed and average distance.

After abamectin exposure, 30 embryos in each group were collected in 1.5 mL tubes added with 500 µL of NP40 containing protease inhibitor cocktail. Then, the embryos were placed on ice and completely lysed using a homogenizer. Total protein was obtained after centrifugation. Enhanced BCA protein detection kit (Beyotime, Shanghai, China) was used to calculate the concentration of the total protein. AChE and ATPase activities were detected using AChE activity assay kit and ATPase assay kit (Nanjing Jiancheng Bioengineering Institute, Nanjing, China), respectively. The experiment of ATPase activities was divided into enzymatic reaction and phosphorus determination reaction. Firstly, according to the manufacturer’s instruction, reagents and samples were added into enzymatic reaction systems, ATPase decomposes ATP into ADP and inorganic phosphorus. Then, the amount of inorganic phosphorus was measured. ATPase activity was measured by absorbance at 660 nm. The detailed protocols of AChE and ATPase activities are shown in Supplementary Materials.

### 4.6. Oxidative Stress Analysis

Ten embryos were collected at 96 hpf, stained with 20 μM of 2,7-dichlorofluorescein diacetate (DCFH-DA) probes and further incubated for 1 h. Then, the embryos were washed, anesthetized and fixed in 0.1% low-melting-point agarose. The embryos were photographed using a fluorescence stereo-microscope (Nikon, Tokyo, Japan). The fluorescence intensity value of ROS was calculated using NIS-element D software (NIS Viewer-5.21.00).

In addition, 30 embryos in each group were collected, then washed three times with embryo culture medium. Total protein was extracted using a homogenizer on ice for 10 min. MDA concentration was measured using MDA assay kit (Nanjing Jiancheng Bioengineering Institute, Nanjing, China). This experiment of MDA was divided into four groups, namely blank tube, standard tube, measuring tube and control tube. According to the manufacturer’s instructions, 10 nmol/mL standard absolute ethanol, other reagents in the kit and glacial acetic acid were added sequentially. Then, each sample was heated at 95 °C for 80 min and cooled with running water. The absorbance value at 532 nm was detected by microplate reader. The activities of SOD and CAT were measured using SOD and CAT assay kits (Nanjing Jiancheng Bioengineering Institute, Nanjing, China), respectively. The experiment of CAT was divided into control tube and assay tube. According to the manufacturer’s instructions, reagents and samples were added into enzymatic reaction systems. The activity of CAT was measured at 405 nm. The experiment of SOD was divided into assay tubes and control tubes. According to the manufacturer’s instructions, tissue homogenate and reagents were added into tubes in turn. The absorbance value was determined at 450 nm using Spectra Max i3x (Molecular Devices, San Jose, CA, USA). The detailed protocols of MDA, SOD and CAT kits are shown in Supplementary Materials.

### 4.7. Acridine Orange (AO) Staining

Apoptosis in zebrafish embryonic brains was analyzed by AO staining [44]. Ten embryos in each group were selected and stained with acridine orange solution (5 mg/L) in darkness for 30 min. The embryos were washed three times with embryo culture medium, anesthetized with tricaine and fixed in 1% agarose. The embryos were photographed using a fluorescence stereo-microscope (Nikon, Tokyo, Japan).

### 4.8. Immunoblotting

Zebrafish ZF4 cells were treated with trypsin, seeded into six-well plates, and avermectin infection was performed after the cells grew to 70–80%. After 24 h of incubation, the medium was discarded, PBS was washed twice and 100 µL of NP-40 lysate was added to lyse for 30 min and transferred to an EP tube. Centrifugation was performed at 12,000× *g* at 4 °C for 10 min, then the supernatant was taken in a clean EP tube, 25 µL 5 × SDS loading buffer was added, and it was boiled at 95 °C for 10 min; samples were collected for Western blot detection. GAPDH was used as an internal control, and the protein expression level of apoptosis gene was detected by Western blotting.

### 4.9. TUNEL Staining

Zebrafish ZF4 cells were treated with trypsin and seeded into confocal dishes, and avermectin infection was performed after the cells grew to 70–80%. After 24 h of incubation, the medium was discarded, PBS was washed twice and the cells were fixed with cell fixative solution for 15 min. Triton-X100 was then permeabilized, PBS was washed twice and the cells were treated according to the manufacturer’s instructions using the TUNEL BrightGreen Apoptosis Detection Kit (Vazyme, Nanjing, China); apoptosis was analyzed by confocal microscopy measurements.

### 4.10. Statistical Analysis

The data from qRT-PCR, morphological analysis and enzymatic determination were calculated by GraphPad Prism 8.0 and presented as mean ± SD. The significant differences were analyzed using one-way ANOVA test followed by Dunnett’s multiple comparisons tests (* *p* < 0.05, ** *p* < 0.01 and *** *p* < 0.001).

## 5. Conclusions

In conclusion, abamectin can cause severe neurotoxicity in zebrafish embryos. In detail, the brain development was impaired upon exposure to abamectin in zebrafish embryos, which was determined using HE staining, locomotor behavior assessment and qRT-PCR analysis. Moreover, following exposure to abamectin, oxidative stress was triggered by the increased level of ROS, and the activities of antioxidant enzymes were inhibited, eventually leading to neuroapoptosis in the zebrafish embryonic brain.

## Figures and Tables

**Table 1:** Sequences of the qRT-PCR primers used in this study.

Target Genes	Prime Sequences (5′-3′)	Accession Numbers
*β-actin-F*	AGCACGGTATTGTGACTAACTG	AF057040.1
*β-actin-R*	TCGAACATGATCTGTGTCATC	
*Bax-F*	TCGAACATGATCTGTGTCATC	AF231015.1
*Bax-R*	TATGGCTGGGGTCACTTTTCTC	
*Bcl2-F*	TGGCGTCCCAGGTAGATAAT	AY695820.1
*Bcl2-R*	ACCGTACATCTCCACGAAGG	
*p53-F*	CCCGGATGGAGATAACTTG	U60804.1
*p53-R*	CACAGTTGTCCATTCAGCAC	
*casepase3-F*	GAGACCGCTGCCCATCACTAG	NM_131877.3
*casepase3-R*	ATCCTTTCACGACCATCT	
*IL-8-F*	TTTCAGCCTTCATGCTTCT	HF674400.1
*IL-8-R*	AGTCACCTTCAGTCCGAGTA	
*apaf1-F*	CCCTCTGTCCAGGCGATTC	AF251502.1
*apaf1-R*	GCCAGCCATGCCAAATACA	
*sox10-F*	GAACGGGTACGACTGGACG	AF402677.1
*sox10-R*	AATGCGATTGGCTGTGGC	
*nestin-F*	AGAAGGTCGGTCAACTCG	XM_001919887.7
*nestin-R*	GGTCTGGGATGCTGGTAG	
*gap43-F*	ACGCCTCCACAGAAACAC	NM_131341.1
*gap43-R*	GCTGCGGCTCCTTCACTT	
*abat-F*	CTGATAGTGAAGTGGAGGCA	NM_201498.2
*abat-R*	TGAAGAAGTCTGGTGAGGC	
*gad1b-F*	GACCCAAACACGGCTAAT	NM_194419.1
*gad1b-R*	TGAGGATCTCCACCACTTC	
*glsa-F*	CCCAGCGGTCTTCGTTTC	NM_001045044.1
*glsa-R*	CTGCGTTGCTTACACCTTGC	
*gria2b-F*	TTTCCTGGTCAGTCGTTT	NM_001045044.1
*gria2b-R*	GCCAAGTTAGCCGTGTAG	
*grin1b-F*	ACGAGCCCAGTACATAGAG	NM_001144131.1
*grin1b-R*	ACATAGCGGAGGATAAGG	

## Data Availability

The raw data supporting the conclusions of this article will be made available by the authors on request.

## Supplementary Materials

The following supporting information can be downloaded at: https://www.mdpi.com/article/10.3390/ijms26010349/s1.
