# Supplementary material for: Abamectin Causes Neurotoxicity in Zebrafish Embryos
Source: Int J Mol Sci. 2025 Jan 3;26(1):349. doi: 10.3390/ijms26010349 (PMC11719719; doi:10.3390/ijms26010349)
Supplement: Supplementary file 1 [file ijms-26-00349-s001.zip › ijms-3370735-supplementary.pdf]

Supplementary Materials

Figure S1

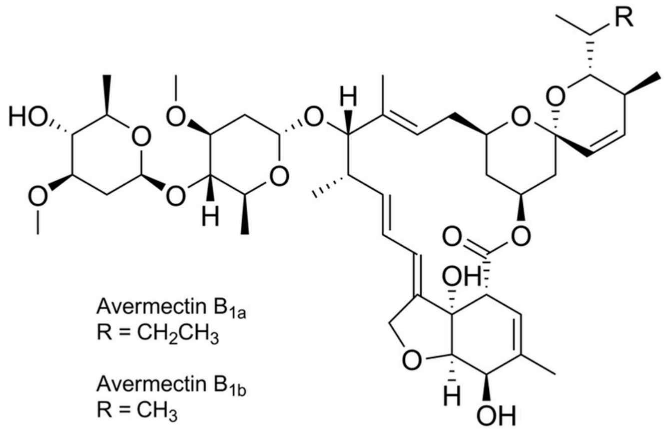

Figure S1 The molecular structure of Abamectin. The currently commercialized avermectin pesticide uses abamectin as the main insecticidal ingredient (avermectin B_{1a} + B_{1b}, of which B_{1a} is not less than 90% and B_{1b} is less than 5%). Abamectin is calibrated based on the content of B_{1a}.

Figure S2

concentration (mg/L) \ Exposure time	Survival Rate(%)											
	24h			48h			72h			96h		
0	90	96.66667	95	90	85	95	90	83.33333	93.33333	90	83.33333	90
0.055	91.66667	93.33333	93.33333	88.33333	85	90	86.66667	86.66667	83.33333	75	70	78.33333
0.0825	90	91.66667	88.33333	86.66667	86.66667	83.33333	71.66667	66.66667	61.66667	61.66667	63.33333	60
0.11	91.66667	88.33333	88.33333	83.33333	80	80	50	48.33333	51.66667	43.33333	41.66667	45
0.1375	88.33333	90	86.66667	73.33333	76.66667	71.66667	33.33333	31.66667	28.33333	18.33333	15	21.66667
0.165	88.33333	85	88.33333	66.66667	68.33333	73.33333	23.33333	28.33333	18.33333	0	0	0

Figure S2 LC50 for zebrafish embryo exposed to abamectin at 96 hpf was 0.11 mg/L.

AChE and ATPase activity

AChE activity assay kit: firstly, the zebrafish was weighted accurately and added 9 times volume of physiological saline to prepare 10% homogenate, then the sample was centrifugated at 3500 rpm for 10 minutes, the protein concentration of the

supernatant was determined by Coomassie brilliant blue reagent.

Operation table:

	**Assay**	**Contrast**	**Standard**	**Blank**
Sample (mL)	a*			
1 μmol/mL standard working solution (mL)			a*	
Distilled water (mL)				a*
Substrate buffer (mL)	0.5	0.5	0.5	0.5
Chromogenic agent working solution (mL)	0.5	0.5	0.5	0.5
Mix sufficiently, react at 37°C for 6 minutes accurately.				
Inhibitor（mL）	0.03	0.03	0.03	0.03
Clarificant (mL)	0.1	0.1	0.1	0.1
Sample (mL)		a*		
The samples were completely mixed, placed for 15 minutes, then transferred into cuvettes of 0.5 cm light path. OD values of all tubes were measured at 412 nm (adjust zero by distilled water).				

Finally, the AChE activity was calculated according to the formula. The calculation formula: [(Determination OD value - control OD value) / (Standard OD value - blank OD value)] × Standard concentration (1 μmol/ml)/Tissue homogenate protein concentration (mgprot/ml).

ATPase assay kit:

The experiment was divided into enzymatic reaction and phosphorus determination reaction. Firstly, according to the manufacturer's instructions, reagents and samples were added into enzymatic reaction systems, ATPase decomposes ATP into ADP and inorganic phosphorus. Then the amount of inorganic phosphorus was measured. ATPase activity was calculated at 660 nm using microplate reader. The formula: [(Determination of OD value - control OD value)/Standard OD value] × Phosphorus standard concentration × 2.5 × 60 / Reaction time (10 minutes)/Tissue homogenate protein concentration.

Oxidative stress analysis

CAT assay kit: The experiment was divided into control tube and assay tube.

according to the manufacturer's instructions, reagents and samples were added into enzymatic reaction systems, the activity of CAT was calculated using a microplate reader at 405 nm. The formula: (measured OD value - control OD value) × 271/60 / sample size (0.025 ml)/ protein concentration of the sample (mgprot/ml).

MDA assay kit: The trace malondialdehyde content in tissue samples was detected by the malondialdehyde assay kit, a lipid peroxide degradation product, can be condensed with thiobarbituric acid to form a red product. This experiment was divided into four groups, including blank tube, standard tube, measuring tube and control tube. According to the manufacturer's instructions, 10 nmol/mL standard, absolute ethanol and other reagents in the kit and glacial acetic acid were sequentially added, and then heated at 95°C for 80 minutes. Then reagents were cooled with running water. The absorbance value at 532 nm was detected using microplate reader. The contents of MDA were calculated according to the formula. The formula: [(Measured OD value - control OD value) / (Standard OD value - blank OD value)] × Standard concentration (10 nmol/mL)/Protein concentration of the sample to be measured (mgprot/mL).

SOD assay kit: This experiment was divided into assay tube and control tube. According to the manufacturer's instructions, the tissue homogenate and the reagent in the kit were sequentially added.

Well<br>Reagent	Contrast well	Contrast blank well	Sample well	Sample blank well
Sample	-	-	20 μL	20 μL
Double distilled water	20 μL	20 μL	-	-
Enzyme working	20 μL		20 μL	-
Enzyme diluent	-	20 μL	-	20 μL
Substrate working [illegible]	200 μL	200 μL	200 μL	200 μL
The samples were mixed completely, incubated at 37°C for 20 minutes. The enzyme-labeled instrument was used to measure absorbance values at 450 nm.				

The activity of SOD was calculated according to the formula. The formula: [(Measured OD value - Measured blank OD value) - (control OD value - control blank OD value)] / (control OD value - control blank OD value)/50% × 0.24 ml / 0.02 ml /

Protein concentration of the sample to be measured (mgprot/mL).
